# Interaction of Isocitrate Lyase with Proteins Involved in the Energetic Metabolism in *Paracoccidioides lutzii*

**DOI:** 10.3390/jof6040309

**Published:** 2020-11-23

**Authors:** Kleber Santiago Freitas e Silva, Raisa Melo Lima, Patrícia de Sousa Lima, Lilian Cristiane Baeza, Roosevelt Alves da Silva, Célia Maria de Almeida Soares, Maristela Pereira

**Affiliations:** 1 Laboratório de Biologia Molecular, Instituto de Ciências Biológicas, Universidade Federal de Goiás, Goiânia 74690-900, Goiás, Brazil; raisamelolima@hotmail.com (R.M.L.); pathricialima@gmail.com (P.d.S.L.); lilianbaeza@gmail.com (L.C.B.); cmasoares@gmail.com (C.M.d.A.S.); 2 Laboratório de Microbiologia Experimental, Centro de Ciências Médicas e Farmacêuticas, Universidade Estadual do Oeste do Paraná, Cascavel 85819-170, Paraná, Brazil; 3 Núcleo Colaborativo de Biossistemas, Instituto de Ciências Exatas, Universidade Federal de Jataí, Jataí 75801-615, Goiás, Brazil; rooseveltfisicaufg@gmail.com

**Keywords:** protein–protein interaction, *Paracoccidioides*, central metabolism, isocitrate lyase

## Abstract

Background: Systemic mycosis is a cause of death of immunocompromised subjects. The treatment directed to evade fungal pathogens shows severe limitations, such as time of drug exposure and side effects. The paracoccidioidomycosis (PCM) treatment depends on the severity of the infection and may last from months to years. Methods: To analyze the main interactions of *Paracoccidioides lutzii* isocitrate lyase (ICL) regarding the energetic metabolism through affinity chromatography, we performed blue native PAGE and co-immunoprecipitation to identify ICL interactions. We also performed in silico analysis by homology, docking, hot-spot prediction and contact preference analysis to identify the conformation of ICL complexes. Results: ICL interacted with 18 proteins in mycelium, 19 in mycelium-to-yeast transition, and 70 in yeast cells. Thirty complexes were predicted through docking and contact preference analysis. ICL has seven main regions of interaction with protein partners. Conclusions: ICL seems to interfere with energetic metabolism of *P. lutzii*, regulating aerobic and anaerobic metabolism as it interacts with proteins from glycolysis, gluconeogenesis, TCA and methylcitrate cycles, mainly through seven hot-spot residues.

## 1. Introduction

The genus Paracoccidioides comprises an intricate group of five phylogenetic species: Paracoccidioides brasiliensis, Paracoccidioides lutzii, Paracoccidioides americana, Paracoccidioides venezuelensis and Paracoccidioides restrepiensis [1,2]. They are the causative agents of paracoccidioidomycosis (PCM), which is a neglected human systemic mycosis. Paracoccidioides is thermodimorphic, the yeast form produces multi-budding cells at 37 °C and the mycelium infecting form grows at 22 °C [3].

The inhalation of fungal propagules is the first step of the infection that takes place in the lungs, where the parasite turns into its pathogenic yeast-like form [4]. The transition from mycelium to yeast form also takes place in vitro after a temperature shift. The thermodimorphism is governed by a set of molecular changes which have been investigated through transcriptomic and proteomic approaches [5,6]. Macrophages in the alveolar tissue is the first defense mechanism fought by the pathogen. Infection success depends on *Paracoccidioides*’ ability to evade oxidative stress generated by the harsh environment inside macrophages [7].

Systemic mycosis is an important cause of morbidity and mortality, especially against immunocompromised subjects [8]. The treatment directed to evade fungal pathogens shows severe limitations, such as time of drug exposure and side effects [9]. The PCM treatment depends on the severity of the infection and may last from two months, in simple cases, to even years in more complex cases. Therefore, there is a real necessity of finding new potential antifungal drugs. Several natural compounds have been tested against *Paracoccidioides* and they have shown promising results [10,11,12,13].

Enzymes from the glyoxylate cycle are related to virulence in a large variety of pathogens [14] and inhibitors have been tested against them, such as *Candida albicans* [15,16], *Mycobacterium tuberculosis* [17,18] and *P. lutzii* [11]. The ICL enzyme is expressed in organisms of several kingdoms, bacteria and fungi [19], protozoa [20], algae [21] and plants [22]. Developing inhibitors against the specific enzymes from the glyoxylate cycle is very promising since malate synthase (MLS) and isocitrate lyase (ICL) are not found in humans. Therefore, a possible drug based on their structures would be less toxic to human cells. ICL is a metabolic enzyme important to microorganisms’ growth based on fatty acids, which is fed by anaplerotic reactions from the glyoxylate shunt. The enzyme is also related to the metabolism of propionyl CoA produced by β-oxidation. In addition, deletion of the ICL gene prevets microorganisms to incorporate carbon from fatty acids [23].

Research on molecular biology and related fields has shed light on the understanding of molecular mechanisms related to protein function. Yet, proteins usually exert their functions at cellular levels through interactions with other proteins or molecules [24]. For the investigation of protein–protein interactions (PPIs), its biological context needs to be taken into account. A specific, transient interaction occurs depending on the cell state, protein modification processes, cell phase, presence of a cofactor or the environment where the cells are [25]. Identified proteins by PPI methodologies are normally isolated from the soluble extract, which contains proteins from different cellular compartments. We did not find any studies related to sub-cellular compartments where ICL is expressed in *Paracoccidioides*. For future perspectives, the identification of the sub-cellular compartments where ICL is present could help to validate more interactions of this enzyme. Regarding model organisms, ICL is present in the extracellular matrix [26], vacuoles [27] and cytoplasm [28,29]. 

Here, we analyzed the main PPIs stablished by ICL in three different *P. lutzii* phases, mycelium, mycelium-to-yeast transition and yeast regarding the energetic metabolism. The PPIs experiments were performed by affinity chromatography, blue native PAGE (BN-PAGE) and validated by co-immunoprecipitation. In addition, we conducted in silico approaches in order to analyze the interface of interaction between ICL and target proteins. ICL has seven main conserved points of interaction with the 15 selected proteins. The residue 505 is the region with the most preferable area of interaction. Thus, ICL may interfere with the central energetic metabolism of *P. lutzii* that could regulate aerobic and anaerobic metabolism, as it interacts with proteins from glycolysis, gluconeogenesis, tricarboxylic acid cycle (TCA) and methylcitrate cycles, mainly through seven hot-spot residues.

## 2. Materials and Methods

### 2.1. P. lutzii Growth Conditions

The *P. lutzii* (ATCC-MYA-826) isolate was used to perform all of the experiments described here. The fungus was maintained on Fava-Netto solid medium (1.0% *w*/*v* peptone, 0.5% *w*/*v* yeast extract, 0.3% *w*/*v* proteose peptone, 0.5% *w*/*v* beef extract, 0.5% *w*/*v* NaCl, 4% *w*/*v* glucose, and 1.4% *w*/*v* agar, pH 7.2) [30]. Mycelium cells grew at 22 °C and were collected on the 12th day of growth. Yeast cells grew at 37 °C and they were collected on the 3rd day of growth. To perform protein extraction, mycelium cells were grown in Fava-Netto liquid medium for 72 h at 22 °C and then mycelium-to-yeast transition was induced by temperature shift from 22 °C to 37 °C [4]. The growth was tracked through a Neubauer chamber and cells were retrieved four days after the temperature shift was established in order to perform protein extraction.

### 2.2. Preparation of P. lutzii Protein Extracts

The protein extraction was methodologically performed equally for mycelium, mycelium-to-yeast transition and yeast cells. *P. lutzii* cells were retrieved by centrifugation at 10,000× *g* for 15 min at 4 °C and washed in sterile phosphate buffered saline (PBS; 1.4 mM KH_2_PO_4_, 8 mM Na_2_HPO_4_, 140 mM NaCl, 2.5 mM KCl at pH 7.2). The cells were resuspended in 20 mM Tris–HCl at pH 8.8 and 2 mM CaCl_2_. Protease inhibitor was added to avoid protein degradation (GE Healthcare, Uppsala County, Uppsala, Sweden). The samples were subjected to cell disruption through five cycles of 30 s each, using a bead beater equipment (BioSpec, Bartlesville, OK, USA). Samples were centrifuged at 10,000× *g* for 15 min at 4 °C and the supernatant was collected to determine protein concentration through the Bradford (Sigma Aldrich, St. Louis, MI, USA) method. 

### 2.3. P. lutzii Recombinant ICL Expression and Purification

The plasmid bearing the cDNA encoding ICL was obtained from [31] and transformed into *Escherichia coli* BL21 C43 (DE3) cells. ICL expression was induced with 1 mM of isopropyl thio-b-D-galactoside (IPTG; Sigma Aldrich, St. Louis, MI, USA). Cells were collected by centrifugation at 10,000× *g* for 15 min at 4 °C. The cells were resuspended in 20 mM Tris–HCl at pH 8.8 and 2 mM CaCl_2_, then incubated with lysozyme and eventually lysed by sonication. The sample was centrifuged at 10,000× *g* for 15 min at 4 °C. The supernatant with the soluble protein fraction was retrieved and applied to a nickel-nitrilotriacetic acid resin (Ni-NTA; Invitrogen, Carlsbad, CA, USA). For 1 mL of lysate, 250 µL of resin were used. The soluble protein fraction was incubated with the resin for 2 h. Next, the resin was washed five times with native wash buffer (50 mM Na_2_HPO_4_, 20 mM imidazol, pH 8.0) to clean non-specific interactions. The recombinant ICL bound to the resin was eluted with salty buffer (50 mM Na_2_HPO_4_, 250 mM imidazol, pH 6.0) and ICL concentration was measured by the Bradford protocol [32].

### 2.4. Protein Co-Precipitation

Recombinant ICL was used as a bait protein for the chromatographic approach. In the first step, recombinant ICL was immobilized into the Ni-NTA resin. Next, soluble fraction of the protein extracts from mycelium, mycelium-to-yeast transition and yeast was incubated in the Ni-NTA system with ICL for 2 h. The bait-and-prey systems were washed and eluted in order to undergo tryptic digestion. The control was prepared with the incubation of *P. lutzii* protein extract with Ni-NTA resin. Proteins identified in both systems (Ni-NTA + ICL + protein extract and Ni-NTA + protein extract) were excluded from the results.

### 2.5. Blue Native PAGE

BN-PAGE assay was performed according to a protocol stablished previously [33] with some modifications. Soluble total protein extracts from mycelium, mycelium-to-yeast transition and yeast were used in the experiments. The samples were dissolved in 10% (*w*/*v*) glycerol and 50 mM Bis-Tris/HCl at pH 7.0. A 5–18% (*w*/*v*) polyacrylamide gradient gel was casted and a gel buffer (150 mM Bis-Tris/HCl, 1.5 M aminocaproic acid at pH 7.0), a cathode buffer (50 mM tricine, 15 mM Bis-Tris/HCl, 0.02% Coomassie blue G-250, at pH 7.0) and an anode buffer (50 mM Bis-Tris/HCl at pH 7.0) were used to conduct the native electrophoresis. The procedure was performed on a vertical Hoefer SE600 ruby apparatus (GE Healthcare) at 15 °C, with a starting voltage of 150 V until the loaded samples were inside the stacking gel, then a constant current limited to 15 mA and a voltage of 300 V were applied.

### 2.6. Sample Digestion, LC–HDMS^E^ Analysis and Data Acquisition

The pull down eluted from each sample was submitted to concentration and subsequently washed with 50 mM ammonium bicarbonate through a 10-kDa molecular weight cut off in an ultracel-regenerated membrane (Amicon Ultra centrifugal filter, Millipore, Bedford, MA, USA). Protein concentrations were determined via Bradford assay. Equimolar amounts of proteins from each sample (150 μg) were digested and prepared for nanoESI-HDMS^E^ (Nano Electrospray High-Definition Mass Spectrometry) analysis and acquisition, as previously described [34], with some modifications. Qualitative and quantitative 2D nanoUPLC coupled to nanoESI-HDMS^E^ experiments were conducted using 60 min reversed-phase (RP) acetonitrile (0.1% *v*/*v* formic acid) gradients (7–40% (*v*/*v*) at 500 nL/min on a nanoACQUITY UPLC 2D RP × RP Technology system. A RP XBridge BEH130 C18 300 μm × 50 mm, 5 µm nanoflow column (pH 10, first-dimension online fractioning) was used in conjunction with a nanoACQUITY UPLC High-Strength Silica (HSS) T3 75 μm × 15 cm, 1.8 µm column (pH 3, second-dimension analytical runs). Typical on-column sample loads were approximated at 5 mg total protein, containing 150 fmol/µL of an exogenous internal standard digest (rabbit Glycogen Phosphorylase B, accession P00489). For all measurements, the mass spectrometer was operated in resolution mode (20,000 resolution FWHM). All analyses were performed using nanoelectrospray ionization in the positive ion mode nanoESI (+) and a nanoLockSpray (Waters) ionization source.

The mass spectrometer was calibrated with a fragment ion spectrum (MS/MS) of the [Glu1]-fibrinopeptide B (Glu-Fib) doubly charged precursor at *m*/*z* 785.8426 using a 250 fmol/μL solution delivered through the reference sprayer of the NanoLockSpray source at 0.5 µL/min. This precursor was also used for Lockmass correction channel, which was sampled every 30 s. Multiplexed data-independent acquisitions (DIA) with specificity and selectivity of nonlinear “T-wave” ion mobility (HDMS^E^) experiments were performed using a Synapt G2-S HDMS mass spectrometer (Waters), set to switch automatically between low (3 eV) and high-energy (19–45 eV) HDMS Scans (HDMS^E^), applied to the transfer “T-wave” collision-induced dissociation cell filled with argon gas. The quadrupole (MS profile) was adjusted so that the nanoUPLC-HDMS^E^ data were effectively acquired from an *m*/*z* range of 400–2000, which ensured that any masses observed in the high-energy spectra of less than *m*/*z* 400 arose from dissociations in the collision cell.

### 2.7. Data Processing

Mass spectrometry data obtained from nanoESI-HDMS^E^ were processed and searched against the *Paracoccidioides Pb*01 database (http://www.broadinstitute.org/annotation/genome/paracoccidioides_brasiliensis/Multiome.html) using ProteinLynx Global Server (PLGS) version 3.0.2 (Waters, Manchester, UK). Protein identification and quantitative data packaging were performed using dedicated algorithms [35,36]. MS spectra were collected in centroid, de-isotoped and charge-state-reduced mode to obtain associated product ions and a monoisotopic mass for all peptides. Protein identification criteria included: (i) minimum number of fragments ion matches per peptide (2), (ii) minimum number of fragments ion matches per protein (5), (iii) minimum number of peptide matches per protein (1), (iv) maximum protein mass (600 kDa), (v) trypsin was chosen as the primary digest reagent, (vi) carbamidomethylation of cysteine residues as a fixed modification, (vii) methionine oxidation and phosphoryl STY as a variable modification (viii) and a maximum 4% false-positive discovery rate, in at least two out of three technical replicate injections. The cut-off of protein in the identification is according to the number of peptides identified after crossing the results of the identification with the genome of the organism, which is available on Uniprot. The score values are listed in Table 1. The PDBs of the relevant structures that interact with ICL and their significance (C-score confidence and TM-score) used for decoys were added in Supplementary Table S1.

Correct and reversed sequences databases were used to estimate false-positive rates (FPR). Using protein identification replication as a filter, the false-positive rate was minimized because false-positive protein identification, i.e., chemical noise, has a random nature and does not tend to replicate across injections. For the analysis of the protein identification and quantification level, the observed intensity measurements were normalized to the intensity measurement of the identified peptides of the digested internal standard [Glu]^1^-Fibrinopeptide B (GluFib) (Sigma, St. Louis, MO, USA). Peptides and protein tables were generated by ProteinLynx Global Server (PLGS), as previously described [37]. Microsoft Excel (Microsoft, Washington, DC, USA) was used for table manipulations; Uniprot (http://www.uniprot.org) and Pedant on MIPS (http://mips.helmholtz-muenchen.de/funcatDB/) database were used for functional classification; uncharacterized proteins were annotated using NCBI database (https://www.ncbi.nlm.nih.gov/).

### 2.8. Co-Immunoprecipitation

The assay was performed according to [38] with some modifications. Briefly, target antibodies were incubated with protein A sepharose 4B (Invitrogen, Waltham, MA, USA) for 3 h. Then, the solution antibodies and resin were incubated with 3 mg/mL of total protein extract for 3 h. Washing steps were performed with wash solution, 10 m mM tris at pH 7.4, 1% Triton and 1 mM EDTA (ethylenediamine tetraacetic acid). Elution was performed with 0.2 M glycine at pH 2.6 and equal amount of 0.2 M tris at pH 8.0. Eluted samples underwent SDS (sodium dodecyl sulfate)-PAGE followed by Western blot.

### 2.9. STRING Database Analysis

The STRING (Search Tool for the Retrieval of Interacting Genes/Proteins) database maintains PPI data regarding both physical and functional interactions. The tool combines data from a large variety of sources such as textmining, laboratory experiments, co-expression and computational PPI prediction [39]. STRING was used to search for ICL interactions in *P. lutzii* with the highest confidence score (0.900). We used the latest version 11.0 (http://string.embl.de).

### 2.10. 3D Structures Assembly

The 3D structure of *P. lutzii* ICL and its interactors have not been resolved experimentally to this date. Hence, all the amino acid sequences were compared against the PDB using the I-TASSER server (http://zhanglab.ccmb.med.umich.edu/I-TASSER/) [40]. The server performs the modeling on templates based on homology of proteins with experimental structures that are available in the PDB. The quality of the 3D structures was evaluated by the MolProbity server (http://molprobity.biochem.duke.edu/). The model quality evaluation relies on the global and local levels of the analyzed proteins and on the power and sensitivity supplied by the optimized hydrogen placement, atom contact analysis, covalent-geometry and torsion-angle criteria [41]. The root mean square deviation (RMSD), Ramachandran and cluster graphics of the ICL 3D model were published in another approach performed by our group [42].

### 2.11. Molecular Dynamics

Molecular dynamics (MD) simulations were performed using the software GROMACS 4.5.5, AMBER force field (200 ns; Mg^2+^), with explicit solvent (water TIP3P) in order to get a more stable protein structure and most similar to the ICL native conformation. The process solvates the 3D models in a cubic box within the defined force field, tending to improve electrostatic interactions with periodic boundary conditions in all directions of the box [43]. In the first step of the MD, the overall system was balanced through the addition of ions in order to minimize energy variation. Simulations were allowed until the system reached the tolerant limit of 1000 KJ/mol. Any simulation with excessive energy or simulations with atoms that overlap were discarded. The system was then equilibrated through energy relaxation for 100 ps and then simulations were performed at 300 K, 1 atm and time interval of 2 fs without any restriction in the protein conformation.

MD trajectories were assessed every 5 ps and the equilibrium of the trajectories was monitored through RMSD considering non-hydrogen atoms and compared to the initial conformation. The evolution of the overall energy (potential and kinetic altogether) was assessed using GROMACS package [44]. The RMSD graphic shows values from template structures and is used to identify common segments related to structurally conserved regions among simulations, thus the most representative conformational was selected to undergo molecular docking [45].

### 2.12. Molecular Docking

The GRAMM-X protein–protein anchor server (http://vakser.compbio.ku.edu/resources/gramm/grammx/) was used in order to identify the best dual protein complex conformation between *P. lutzii* ICL and interacting proteins [46]. Then, the amino acids involved in the interaction were identified. KFC2 server (https://mitchell-lab.biochem.wisc.edu/KFC_Server/index.php) was used to recognize the contact residues in the interaction interface of ICL and binding proteins [47]. CoCoMAPS (https://www.molnac.unisa.it/BioTools/cocomaps/) server was applied to analyze the interface of the protein complexes through intermolecular contact maps. Thus, it was identified residues of the binding proteins that interact with ICL and it was possible to categorize them as hotspots [48]. The ICL regions most frequently involved in the interactions were proposed based on the most frequent interacting residues.

## 3. Results and Discussion

### 3.1. ICL Interacts with Proteins from the Glycolytic Pathway

**Figure 1:**
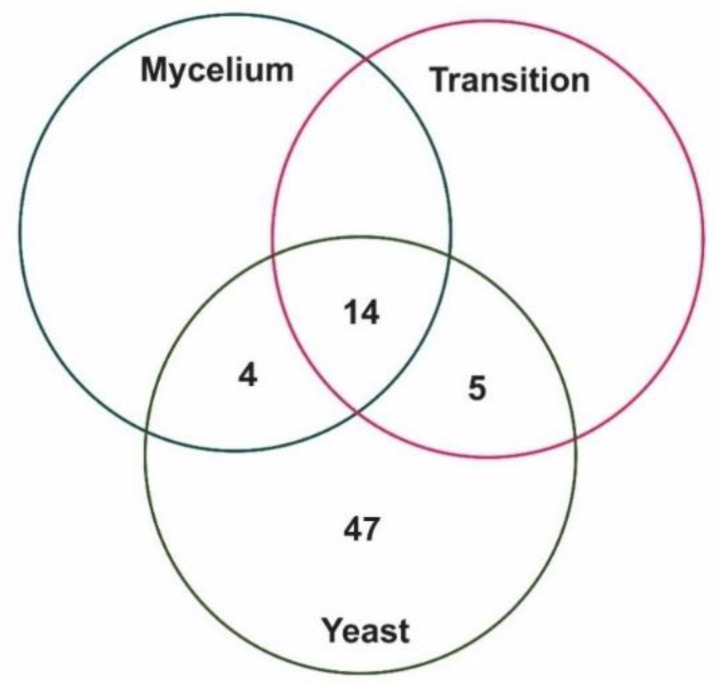
Venn diagram of proteins that interact with ICL. No specific mycelium and mycelium-to-yeast transition proteins were found binding to ICL and 47 proteins were found interacting to ICL specifically in the yeast phase. Yeast proteins are very active in the anaerobic metabolism and ICL interactions may take part in the regulation of both aerobic and anaerobic metabolism.

According to the pull-down assay, ICL interacted with 18 proteins in mycelium, 19 proteins in mycelium-to-yeast transition and 70 proteins related to energetic metabolism in yeast (Table 1). Figure 1 shows the number of proteins that interact with ICL in mycelium, mycelium-to-yeast transition and yeast cells.

**Figure 2:**
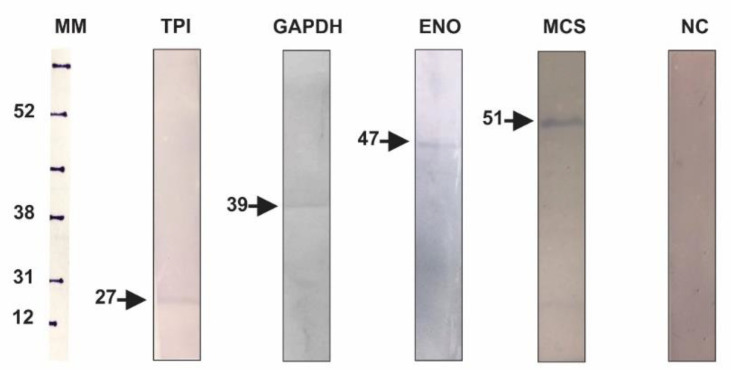
Validation of ICL interacting proteins by the co-immunoprecipitation assay. Here, anti-ICL antibody was incubated with protein A sepharose 4B and then with *P. lutzii* total protein extract. Eluted samples underwent SDS-PAGE followed by Western blot. TPI (triose phosphate isomerase; 27 KDa), GAPDH (glyceraldehyde-3-phosphate dehydrogenase; 39 KDa); ENO (enolase; 47 KDa), MCS (2-methylcitrate synthase; 51 KDa) and NC (negative control). MM—rainbow molecular marker, Amersham™. MCS and GAPDH were identified from the yeast phase, ENO from mycelium-to-yeast transition and TPI from the mycelium phase. The antibodies anti-MCS [49], anti-GAPDH [50], anti-ENO [51] and anti-TPI [52] were produced by our laboratory group. The interaction between ICL and MCS, GAPDH and TPI were identified both by chromatography and BN-PAGE and the interaction between ICL and ENO was identified by BN-PAGE.

Among proteins that bound to ICL in the pull-down assay, phosphoenolpyruvate carboxykinase (PEPCK), 2-methylcitrate dehydratase (2MDH), 2-methylcitrate synthase (MCS), 12-oxophytodienoate reductase (OPR), glyceraldehyde-3-phosphate dehydrogenase (GAPDH), enolase, among others, were validated by the BN-PAGE approach. MCS was also validated by co-immunoprecipitation, along with glyceraldehyde-3-phosphate dehydrogenase, triosephosphate isomerase (TPI) and enolase (ENO) (Figure 2).

**Figure 3:**
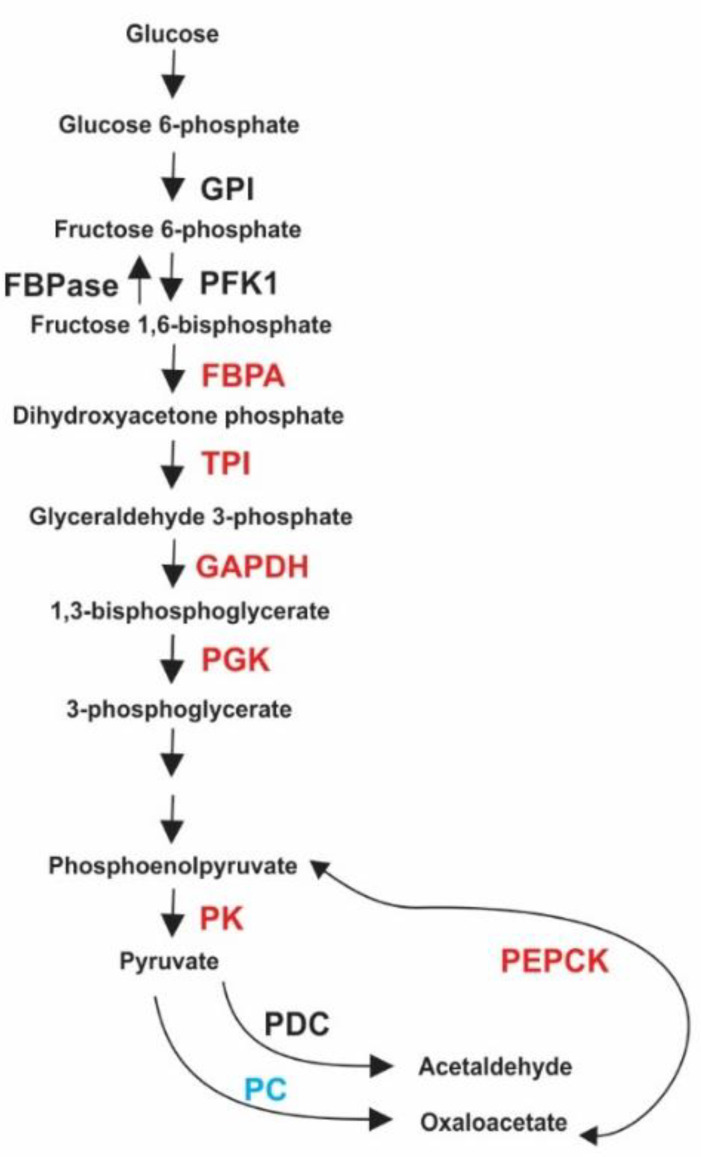
Proteins from glycolysis and gluconeogenesis that interacted with *P. lutzii* ICL. Proteins in black are specific from yeast cells, red refers to proteins that are common to mycelium, mycelium-to-yeast transition and yeast cells. Blue refers to proteins that are common to mycelium-to-yeast transition and yeast cells. No proteins were common to mycelium and mycelium-to-yeast transition. GPI (glucose-6-phosphate isomerase); FBPase (fructose-1,6-bisphosphatase); PFK1 (phosphofructokinase 1); FBPA (fructose-bisphosphate aldolase); TPI (triosephosphate isomerase); GAPDH (glyceraldehyde-3-phosphate dehydrogenase); PGK (phosphoglycerate kinase); PK (pyruvate kinase); PDC (pyruvate decarboxylase); PC (pyruvate carboxylase); PEPCK (phosphoenolpyruvate carboxykinase).

There is a connection among the major energetic metabolic pathways: glycolysis, gluconeogenesis, TCA, glyoxylate and methylcitrate cycles. The expression of certain enzymes from a cycle may inhibit the activity of enzymes from others [53]. In photosynthetic organisms, for example, reactions of the glyoxylate cycle couple with reactions of TCA to feed intermediates into gluconeogenesis. A null ICL mutation in *Chlamydomonas* was performed in order to analyze the effects of ICL absence in carbon metabolism [54]. They realized that ICL deletion decreased the amount of glycolysis and gluconeogenesis enzymes. Here, ICL interacted with fructose-bisphosphate aldolase (FBPA), PEPCK, phosphofructokinase 1 (PFK1), glucose-6-phosphate isomerase (GPI) and pyruvate kinase (PK; Figure 3), the very same enzymes were down-regulated in the absence of ICL expression in *Chlamydomonas*.

ICL interacted with TPI (Figure 3) in all experimental approaches we performed. In addition, this interaction was validated by co-immunoprecipitation (Figure 2). ICL is a potential target for drug design against several microorganisms, including *M. tuberculosis* [55]. Britton and colleagues (2000) worked with a rational drug design based on the structure of *Aspergillus nidulans* ICL. They found that ICL structure has a TPI similar motif and they share similar interaction interfaces [56]. In multiprotein complexes, common interaction partners are prone to interact through similar interfaces which are usually related to a common motif among those proteins [57].

Phosphoglycerate kinase (PGK) and PFK1 (Figure 3) interacted with ICL according to our pull-down assay (Table 1) and the latter is a regulatory protein from the glycolytic pathway. ICL influences the activity of certain glycolytic enzymes [54]. In addition, it has been shown that some products of glycolytic reactions can inhibit ICL activity [58]. The product of PGK activity is 3-phosphoglycerate and fructose-1,6-bisphosphate is the product of PFK1. Both inhibited ICL activity efficiently in *Corynebacterium glutamicum*, showing that these enzymes have a role in controlling the energetic metabolism of cells. Interestingly, ICL may participate in the regulation of energetic metabolism and influence the production or consumption of ATP in pathways, such as glycolysis. We found two glycolytic enzymes that undergo allosteric regulation, PFK1 and PGK [59], and their interaction with ICL may affect *P. lutzii* metabolism driving it either to aerobic (mycelium phase) or anaerobic (yeast phase) conditions.

Phosphoenolpyruvate is produced from pyruvate during gluconeogenesis. First, pyruvate carboxylase (PC) irreversibly converts pyruvate into oxaloacetate, which is then converted into phosphoenolpyruvate by PEPCK (Figure 3). Overexpression of ICL recovered growth of PC mutant cells. They showed that ICL is involved in anaplerotic reactions of phosphoenolpyruvate and oxaloacetate, which are important for gluconeogenesis [60]. Enzymes from the glyoxylate cycle and TCA are required for a proper function of gluconeogenesis [61]. Fructose-1,6-bisphosphatase (FBPase), ICL and PEPCK are secreted in *Saccharomyces cerevisiae* grown in low glucose [62] showing that these proteins take part in the metabolic shift of fungal cells. These experimental pieces of evidence show that the relation between energetic metabolic enzymes are rather intricate and complex, highlighting a great potential of cell metabolism for adaptation to several conditions and perturbations.

There are some similarities between ICL and PEPCK. Both enzymes are essential for certain pathogens, such as *M. tuberculosis*, to grow inside macrophages [63]. ICL influences several metabolic pathways such as glycolysis, gluconeogenesis and TCA, this way the enzyme roles would be more complex than only being related to a metabolic shift from carbohydrates to fatty acids during the host-pathogen interaction [64]. Interestingly, we found *P. lutzii* ICL interacting with proteins from all of those metabolic pathways (Table 1). ICL could play anabolic roles, since its flux reactions do not feed TCA for catabolism but for biosynthesis through the succinyl-CoA node [64]. There is also evidence that ICL and PEPCK influence gluconeogenesis in plants [65,66]. Interaction between ICL, PEPCK and other proteins from glycolysis and gluconeogenesis may be important to regulate central metabolism in *P. lutzii,* since it is well known that in *Paracoccicioidies*, the yeast phase is preferably grown in anaerobic conditions and mycelium phase in aerobic conditions [5].

Figure 3 schematically shows proteins related to glycolysis that interacted with ICL in the pull-down assay and some of the multiprotein complexes ICL forms were validated by BN-PAGE. Here, ICL may play important roles regulating glycolysis and gluconeogenesis. Moreover, ICL interacts with enzymes that are points of regulation of glycolysis such as PFK1 and PK. As ICL is related to a metabolic shift [64], it might as well regulate proteins in these pathways to drive aerobic metabolism to mycelium cells and anaerobic metabolism to yeast cells.

### 3.2. ICL Influences TCA Flux and Electron Chain Dynamics

The ICL activity and its importance to certain pathogens survival and persistence within host cells are related to its role in the glyoxylate shunt, which provides a metabolic shift in the principal carbon source from carbohydrates into fatty acids [67]. The glyoxylate shunt function as an anaplerotic pathway necessary for the metabolism of fatty acids but can also function in more general conditions, such as influencing oxidative reactions of TCA [68].

**Figure 4:**
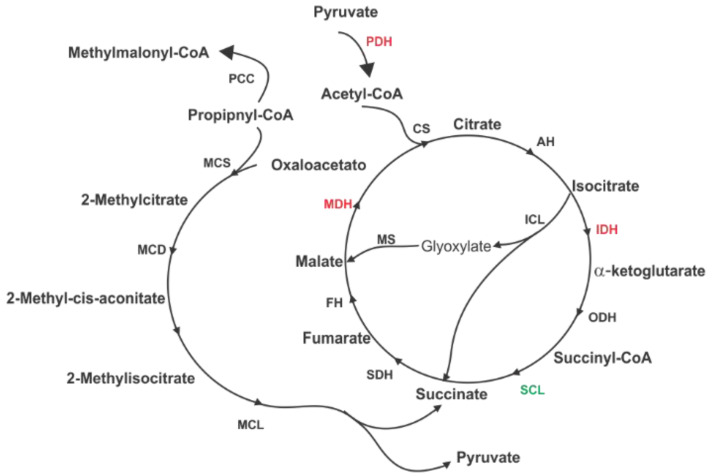
The diagram shows proteins from TCA, glyoxylate shunt and methylcitrate cycle that interacted with ICL. Most of the proteins were identified in the pull down performed on yeast cells (protein in black), only MDH (malate dehydrogenase), IDH (isocitrate dehydrogenase) and PDH (pyruvate dehydrogenase) were found in the pull down of protein extracts from mycelium, mycelium-to-yeast transition and yeast cells (proteins in red). SCL (succinyl-CoA ligase) was found in protein extract from mycelium and yeast cells (proteins in green). CS (citrate synthase); AH (aconitase); ODH (2-oxoglutarate dehydrogenase); SDH (succinate dehydrogenase); FH (fumarate hydratase); PCC (propionyl-CoA carboxylase); MCS (2-methylcitrate synthase); MCD (2-methylcitrate dehydratase); MCL (mitochondrial 2-methylisocitrate lyase).

In an ICL deleted *M. tuberculosis* mutant, TCA intermediates were down-regulated when compared to wild-type cells and they showed accumulation of propionate due to the metabolism of fatty acids [69]. The authors suggested that the absence of ICL might have impaired respiratory processes due to TCA intermediate depletion and alteration of the methylcitrate cycle. Our results show *P. lutzii* ICL interacting with proteins from all of those pathways (Table 1; Figure 4), and we hypothesize that *P. lutzii* ICL might act as a regulator of TCA anaplerotic reactions and it also influences respiratory activities.

The state of fragility regarding accumulation of propionate is not only related to the absence of ICL activity but due to metabolic flaws such as derangement of methylcitrate cycle, less amount of oxaloacetate from the TCA and the gluconeogenic pathways. ICL interacted with mitochondrial 2-methylisocitrate lyase (MCL), MCS and 2-MDH from methylcitrate cycle and with malate dehydrogenase (MDH) from TCA (Table 1 and Table 2; Figure 4). These interactions may help to control the energetic metabolism in pathogen cells to eliminate propionate and maintain a normal level of anaplerotic constituents. Interestingly, accumulation of methylmalonyl-CoA due to partial degradation of propionyl-CoA by propionyl-CoA carboxylase was observed [69] and we found ICL interacting with this protein as well (Figure 4). Finally, studies on the effects of ICL overexpression resulted in a higher degree of oxidative TCA reactions with higher production of succinate, fumarate, citrate and oxaloacetate [70]. *P. lutzii* ICL interacted with enzymes responsible for the production of such intermediates: succinyl-CoA ligase, succinate dehydrogenase, citrate synthase and MDH (Table 1 and Table 2; Figure 4).

TCA and glyoxylate cycles enzymes are under investigation and new functions for such enzymes have been proposed. Isocitrate dehydrogenase (IDH) and ICL, for example, were found to be related to riboflavin production, which also uses TCA intermediates such as malate. Disruption of the genes that encode for IDH and ICL led to a significant decrease in riboflavin levels [71]. Here, ICL interacted with IDH and other proteins from TCA, showing that it may play important roles in central metabolism of *Paracoccidioides*. The relationship between the two specific enzymes of glyoxylate cycle has also been investigated. In *Caenorhabditis elegans* and *Euglena gracilis*, ICL and MLS are expressed as a single bifunctional polypeptide being encoded by a single gene [72,73]. We found ICL interacting with MLS in *P. lutzii* (Table 1) and their interaction may promote substrate channeling, which could raise the concentration of enzymatic intermediates and reduce the concentration of enzyme required to maintain the intermediate flux in that pathway. Most importantly, the final product is driven in a scaffold manner to a specific subcellular location preventing loss of such intermediates [74].

Aconitase (AH) catalyzes the isomerization of citrate to isocitrate. The latter can be used either in the glyoxylate shunt or in TCA by ICL or IDH, respectively. ICL interacted with AH and IDH (Table 1 and Table 2) in the pull-down assay (Figure 4). These proteins play important roles in driving the metabolism of cells towards aerobic or anaerobic conditions. Over the decades, biotechnological studies have attempted to change the level of anaplerotic products produced by microorganisms through deletion of genes involved in the TCA cycle and the glyoxylate shunt due to the involvement of the enzymes from these pathways in the aerobic and anaerobic metabolism [75,76]. The relation of the TCA cycle and the glyoxylate shunt in the production of succinate has been investigated. According to previous knowledge, *S. cerevisiae* cells produce succinate via oxidative reactions of TCA and the glyoxylate shunt. AH and ICL double mutants inhibited oxidative reactions of those pathways. Double mutants showed a slower growth rate compared to control due to defect in catabolizing carbon compounds. The deletion also altered fermentation properties of the cells with lower production of succinate [77]. This is another indication that ICL influences central metabolism and may play important metabolic roles in *P. lutzii* besides participating in the conversion of acetyl-CoA to succinate for the synthesis of carbohydrates.

ICL interacted with several enzymes that take part in the electron transport chain and respiration (Table 1 and Table 2). ICL deletion mutants have anaplerotic intermediates imbalance and the accumulation of certain compounds, such as propionate, as discussed previously. This leads to changes in the NAD/NADH ratio, consequently altering pH and the membrane potential responsible for the generation of intracellular ATP. Some authors have suggested that ICL could play important roles in maintaining the balance between glycolysis, TCA and respiratory chain in order to maintain an energized and functional membrane for energy generating processes [69]. Proteins act within interconnected pathways and networks that evolved over the years in order to be robust and give cells the ability to adapt more and more to the environment. Thus, identifying metabolons and new PPIs might shed light on specific biochemical pathways associated with a given phenotype or perturbation such as the microenvironment of a macrophage.

Figure 4 shows proteins from TCA and methylcitrate cycle that interacted with ICL. The glyoxylate shunt enzymes, mainly ICL, take part in anaplerotic pathways required primarily for the metabolism of fatty acids. They can also affect general conditions, such as the reactions of TCA and consequently the respiratory chain. Here, we hypothesize that ICL might act as one of the regulators of TCA anaplerotic reactions, methylcitrate cycle and respiratory chain.

### 3.3. STRING PPI Database Validates ICL Protein Partners from Pull-Down Assay

We compared our results with ICL binding proteins in the STRING database. According to STRING, 31 proteins interact with *P. lutzii* ICL. The PPI prediction score generated by STRING ranged from 0.600 to 0.998. Among those proteins, 16 interacted with ICL from yeast cells, none in mycelium cells and one in mycelium-to-yeast transition cells (Table 2 and Supplementary Figure S1). There were 15 proteins identified in the STRING databank that were not found in our results, probably because the techniques used might disrupt weak and transient interactions [78].

**Figure 5:**
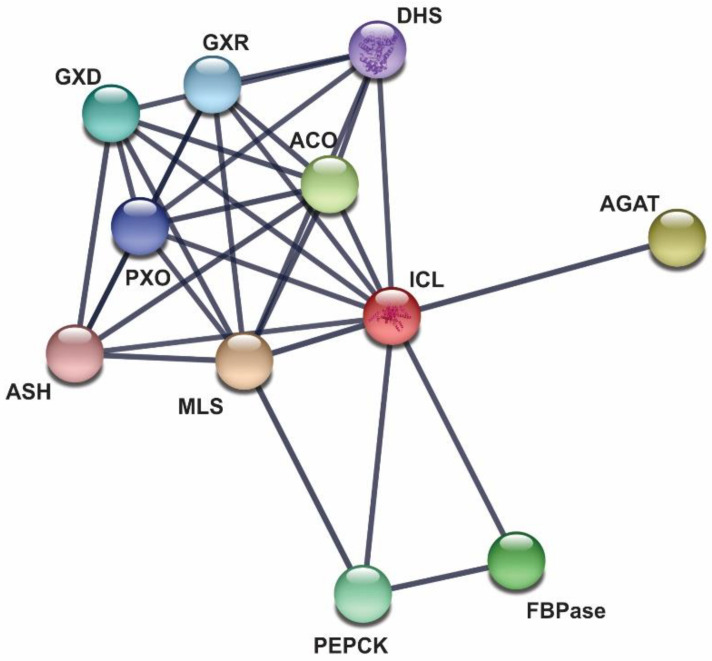
ICL interacting proteins according to STRING database. STRING identified ten *P. lutzii* ICL partners [39] when the score selected was of the highest confidence (0.9). This database collects data textmining, laboratory experiments, co-expression and computational PPI prediction sources. The types of interaction were hypothetical for ASH and DHS and homolog expression for all the other ICL partners identified by STRING. ACO (aconitate hydratase), DHS (dihydrodipicolinate synthase), GXR (glyoxylate reductase), PXO (peroxisomal oxidase), GXD (glyoxylate dehydrogenase), MLS (malate synthase), (PEPCK) phosphoenolpyruvate carboxykinase, FBPase (fructose-1,6-bisphosphatase), ASH (aspartate-4-semialdehyde hydrolase) and AGAT (alanine-glyoxylate aminotransferase).

Only four proteins showed scores higher than 0.900 and among them 3-isopropylmalate dehydratase large subunit (IMDH) and MLS (Figure 5) were found in our experimental approaches. Interestingly, all proteins bound to ICL either in mycelium or in mycelium-to-yeast transition phases were also bound to ICL in the yeast phase according to STRING. To our knowledge, no study has been performed focusing on the comparison of PPIs in different phases of dimorphic microorganisms.

### 3.4. P. lutzii ICL Has Several Regions of Interaction Interface

The KFC-2 server provided means to identify the most common residues involved in the interaction of ICL with other proteins. Finding hot spots on the protein surface has shed light on experimental applications in the biological fields [79,80,81] and understanding PPIs means understanding proteins’ biological functions. Here, we used this approach in order to identify the main contact preference regions of *P. lutzii* ICL when it interacted with proteins from mycelium, mycelium-to-yeast and yeast phases. Moreover, the technique has been applied for different purposes such as rational drug design [79], understanding the physiology of transient PPIs [82] and the understanding of specific virulence mechanisms in pathogens [83].

**Figure 6:**
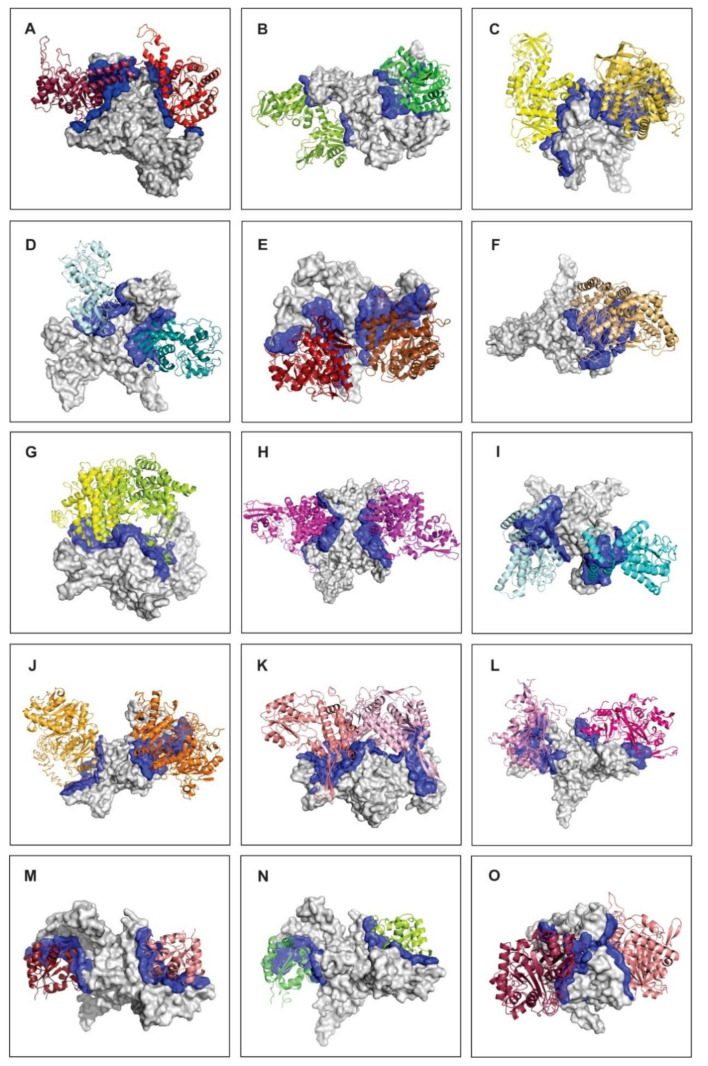
Schematic representation of *P. lutzii* ICL interactions with 15 metabolic protein partners. Each image shows two conformations of *P. lutzii* ICL (gray), its protein partners (different colors) and the interacting interface (dark-blue). We chose the best two conformations of the complexes with the lowest free energy in order to represent the most probable regions of ICL interaction with other proteins. (**A**) fructose-bisphosphate aldolase (FBA1); (**B**) phosphoglycerate kinase; (**C**) pyruvate kinase; (**D**) glyceraldehyde-3-phosphate dehydrogenase; (**E**) enolase; (**F**) malate dehydrogenase; (**G**) 2-methylcitrate synthase; (**H**) 2-methylcitrate dehydratase; (**I**) enoyl-CoA-hydratase (ECH); (**J**) phosphoenolpyruvate carboxykinase; (**K**) alcohol dehydrogenase (ADH); (**L**) fumarylacetoacetase (FAA); (**M**) ribose-phosphate pyrophosphokinase (RPP); (**N**) nucleoside diphosphate kinase (NDK); (**O**) acetyl-CoA acetyltransferase (ACA). (**A**–**D**,**F**–**H**,**J**) interacted with ICL in *P. lutzii* protein extracts in the chromatographic assay and (**E**,**I**,**K**–**O**) interacted with ICL in *P. lutzii* protein extracts in the BN-PAGE assay.

**Figure 7:**
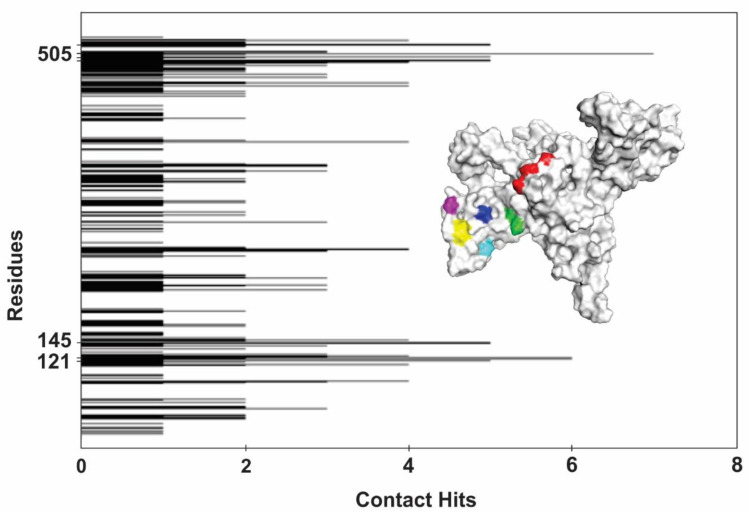
Contact preference residues of *P. lutzii* ICL interacting with metabolic proteins. The contact preference analysis showed the frequency of interaction between ICL amino acid residues and its selected metabolic protein partners. ICL region around residue 505 is the most energetically favored to interact with amino acids from ICL-binding proteins with 5–7 hits of interaction and region around residue 121 with 5–6 hits. The three-dimensional structure shown here highlights the seven residues that interact with ICL partners most frequently. The colors represent the amino acids scored as hot spots, MET 120 and ASN 121 (red), GLN 502 and LYS 503 (green), SER 505 (dark blue), LYS 513 (pink), SER 525 and ARG 526 (yellow) and VAL 530 (light blue).

We selected 15 proteins for this in silico analysis. Among them, 10 ICL binding proteins related to central metabolism (Figure 6A–J) since ICL interferes with it in a variety of pathogens [54,64]. Proteins from secondary metabolism formed the other complexes (Figure 6K–O). This selection was based on their relevance for the *Paracoccidioides* metabolism. For each ICL binding protein, we generated two models of interaction featuring the two most stable conformations of the complex. Through the *P. lutzii* ICL docking analysis and the contact preference regions analysis, 30 complexes have been proposed (Figure 7).

We found seven ICL regions that interacted with the selected protein partners. Figure 7 highlights the seven main regions that participate in the *P. lutzii* ICL interactions. The small colored areas are regions with residues that most frequently interact with the 15 selected proteins for this in silico analysis. Figure 6 highlights the interface of interaction between ICL and its binding proteins, those contact amino acids are spread around that interface region. All ICL partners interacted with SER 505, which seems to be the most important hot spot that energetically contributes to the stabilization of the binary complex conformation. MET 120 and ASN 121 are contact residues between ICL and FBA1, PGK, PK, GAPDH and ENO. The fact that those proteins belong to the glycolytic pathway and since they share similar contact residues with ICL, reinforce the idea of the existence of a glycolytic metabolon in *P. lutzii* [84]. GLN 502 and LYS 503 establish contact between ICL and partners in ENO, MDH, MCS and MCD, while LYS 513 of ICL interacts with PK, MDH, ECH and ACA. The residues SER 525 and ARG 526 are present in the interface of interaction binding to FBA1, PEPCK, ADH, NDK and ACA. Finally, the residue VAL 530 interacts with PGK, MCS, ADH, FAA and RPP.

### 3.5. The Amino Acid Residue 505 of ICL Belongs to a Very Active Binding Region

The availability of big data post-high-throughput era provides means to predict networks of PPIs. An interesting approach regarding contact preference regions categorized PPI complexes available in Swissprot and PDB showed that certain amino acids are preferred for specific types of interfaces [85]. Hydrophobic interactions are the most important forces within the contact preference regions [86] and the most frequent residues that interacted with ICL partners were hydrophobic.

The graphic in Figure 7 represents the contact preference regions of *P. lutzii* ICL and its protein partners. The graphic points out that the ICL binding region near residue 505 is the region with the most preferable area of interaction. The area near residues 120, 121, 145, 502, 503 and 529 are also very active and most of the protein partners of *P. lutzii* ICL interact with residues on these contact preference regions.

## 4. Concluding Remarks

We performed three different assays of PPIs in order to establish the protein partners of ICL. ICL may interfere with central metabolism of *P. lutzii*, regulating aerobic and anaerobic metabolism in mycelium, mycelium-to-yeast transition and yeast cells as it interacts with proteins from glycolysis, gluconeogenesis, TCA and methylcitrate cycles. The data presented here demonstrate that the function of ICL extends beyond lipid metabolism and that this protein has a role in regulating anaplerotic reactions of central metabolism. ICL has seven regions that interacted with the selected protein partners more frequently. The residue 505 is the region with the most preferable area of interaction.

## 5. Future Perspective

The PCM treatment presents limitations, such as administration of toxic antifungals for an extended period. The search for new potential antifungals is extremely relevant. Studies on PPIs have led to the findings of new antifungal compounds and the design of promising small molecules (peptides) also with antifungal properties. This is the future perspective of the innovative study presented here. In this work, we describe the PPI profile of *P. lutzii* ICL with metabolic proteins. The results presented here will provide ways of screening new ICL inhibiting compounds and the design of ICL modulator peptides to be tested as anti-PCM. ICL is a good target for PCM new therapies, because this enzyme is absent in humans and has been described as an important virulence factor to several pathogens.

## Figures and Tables

**Table 1:** Proteins related to central metabolism binding to ICL in the pull-down assay in *P. lutzii* mycelium, mycelium-to-yeast transition and yeast forms.

Accession	Protein Name	Score **
	***Mycelium***	
	**Glycolysis and Gluconeogenesis**	60,211.38
PAAG_01995	fructose-bisphosphate aldolase	11,197.06
PAAG_02585	triosephosphate isomerase *	29,783.99
PAAG_02869	phosphoglycerate kinase	37,493.65
PAAG_06380	pyruvate kinase	24,097.65
PAAG_08203	phosphoenolpyruvate carboxykinase	10,288.74
	**Tricarboxylic-acid pathway**	18,022.91
PAAG_01534	pyruvate dehydrogenase E1 component subunit beta	54,603.02
PAAG_03330	dihydrolipoyl dehydrogenase	18,252.14
PAAG_00417	succinyl-CoA ligase subunit alpha	15,495.53
PAAG_01463	succinyl-CoA ligase subunit beta	45,050.75
PAAG_07729	isocitrate dehydrogenase subunit 2	29,021.65
PAAG_00053	malate dehydrogenase	64,147.15
PAAG_08449	malate dehydrogenase	40,336.05
PAAG_05048	3-isopropylmalate dehydratase large subunit	24,897.33
	**Electron transport**	16,888.22
PAAG_04820	ATPase alpha subunit	17,866.72
	**Respiration**	82,884.62
PAAG_06796	cytochrome c oxidase subunit 5b	60,211.38
PAAG_08037	ATP synthase subunit beta	11,197.06
	**Pentose-phosphate pathway**	29,783.99
PAAG_04166	transaldolase	37,493.65
PAAG_04444	transketolase	24,097.65
	***Mycelium-to-yeast transition***	
	**Glycolysis and Gluconeogenesis**	
PAAG_01995	fructose-bisphosphate aldolase	14,297.79
PAAG_02585	triosephosphate isomerase *	12,564.27
PAAG_02869	phosphoglycerate kinase	12,361.49
PAAG_06380	pyruvate kinase	8237.422
PAAG_00726	pyruvate carboxylase	5375.759
PAAG_08203	phosphoenolpyruvate carboxykinase	4991.925
	**Tricarboxylic-acid pathway**	
PAAG_01534	pyruvate dehydrogenase E1 component subunit beta	3429.868
PAAG_07729	isocitrate dehydrogenase subunit 2	4581.662
PAAG_08449	malate dehydrogenase	3811.987
PAAG_00053	malate dehydrogenase	2309.6
PAAG_05048	3-isopropylmalate dehydratase large subunit	3299.345
	**Electron transport**	
PAAG_04820	ATPase alpha subunit	4619.729
	**Respiration**	
PAAG_08037	ATP synthase subunit beta	4555.649
PAAG_00953	NADH-cytochrome b5 reductase	2806.097
PAAG_00173	electron transfer flavoprotein subunit alpha	3309.523
PAAG_02265	mitochondrial F1F0 ATP synthase subunit	14,297.79
	**Pentose-phosphate pathway**	
PAAG_04166	transaldolase	12,361.49
PAAG_04444	transketolase	8237.422
	**Energy conversion and regeneration**	
PAAG_03631	12-oxophytodienoate reductase *	4991.925
	***Yeast***	
	**Glycolysis and Gluconeogenesis**	
PAAG_06526	glucose-6-phosphate isomerase	44,267.03
PAAG_01583	phosphofructokinase 1	30,114.88
PAAG_01995	fructose-bisphosphate aldolase	24,984.16
PAAG_02585	triosephosphate isomerase *	22,256.77
PAAG_08468	glyceraldehyde-3-phosphate dehydrogenase *	20,333.02
PAAG_02869	phosphoglycerate kinase	18,095.4
PAAG_06380	pyruvate kinase	20,228.38
PAAG_02512	pyruvate decarboxylase *	18,851.39
PAAG_02050	pyruvate decarboxylase *	18,717.38
PAAG_00726	pyruvate carboxylase	13,367.75
PAAG_08203	phosphoenolpyruvate carboxykinase	23,108.45
PAAG_02682	fructose-1.6-bisphosphatase	14,274.43
PAAG_07986	phosphofructokinase 2	16,924.33
PAAG_07410	2,3-bisphosphoglycerate-independent phosphoglycerate mutase	15,371.76
	**Tricarboxylic-acid pathway**	
PAAG_00094	pyruvate dehydrogenase kinase	11,769.04
PAAG_01534	pyruvate dehydrogenase E1 component subunit beta	13,649.55
PAAG_08295	pyruvate dehydrogenase E1 component subunit alpha	13,423.09
PAAG_02769	pyruvate dehydrogenase protein X component	14,287.29
PAAG_03330	dihydrolipoyl dehydrogenase	11,642.86
PAAG_08915	dihydrolipoamide succinyltransferase	14,385.67
PAAG_01725	succinate dehydrogenase flavoprotein subunit	9554.287
PAAG_04238	succinate dehydrogenase flavoprotein subunit	9647.347
PAAG_06103	succinate dehydrogenase iron-sulfur subunit	12,727.08
PAAG_00417	succinyl-CoA ligase subunit alpha	7841.01
PAAG_01463	succinyl-CoA ligase subunit beta	9100.539
PAAG_00856	isocitrate dehydrogenase subunit 1	11,681.96
PAAG_07729	isocitrate dehydrogenase subunit 2	10,708.71
PAAG_08351	mitochondrial NADP-specific isocitrate dehydrogenase	13,966.9
PAAG_00053	malate dehydrogenase	10,102.86
PAAG_08449	malate dehydrogenase	8959.159
PAAG_05150	ATP-citrate synthase subunit 1	10,582.24
PAAG_05151	ATP-citrate-lyase	10,356.12
PAAG_08075	citrate synthase	9238.283
PAAG_07843	aconitase	12,099.61
PAAG_05048	3-isopropylmalate dehydratase large subunit *	10,060.89
PAAG_00588	fumarate hydratase	9309.173
PAAG_02732	2-oxoglutarate dehydrogenase E1	10,540.35
	**Electron transport**	
PAAG_03051	NADH-ubiquinone oxidoreductase 20.8 kDa subunit	10,945.87
PAAG_01044	NADH-ubiquinone oxidoreductase 24 kDa subunit	8694.258
PAAG_05031	NADH-ubiquinone oxidoreductase 40 kDa subunit	7284.236
PAAG_05735	NADH-ubiquinone oxidoreductase 49 kDa subunit	8447.485
PAAG_02656	NADH-ubiquinone oxidoreductase 51 kDa subunit	9802.509
PAAG_07791	NADH-ubiquinone oxidoreductase	8821.187
PAAG_08916	LYR family protein	7664.982
PAAG_04820	ATPase alpha subunit	7938.354
	**Respiration**	
PAAG_08037	ATP synthase subunit beta	7580.651
PAAG_05576	ATP synthase gamma chain	6790.638
PAAG_04570	ATP synthase D chain mitochondrial	44,267.03
PAAG_04838	ATP synthase subunit 4	30,114.88
PAAG_02679	vacuolar ATP synthase 98 kDa subunit	24,984.16
PAAG_08966	NADH-ubiquinone oxidoreductase 78 kDa subunit	22,256.77
PAAG_02266	NADH-ubiquinone oxidoreductase 21 kDa subunit	20,333.02
PAAG_00953	NADH-cytochrome b5 reductase	18,095.4
PAAG_08088	cytochrome b-c1 complex subunit 2	20,228.38
PAAG_08057	cytochrome c oxidase polypeptide V	18,851.39
PAAG_06796	cytochrome c oxidase subunit 5b	18,717.38
PAAG_03292	cytochrome c peroxidase	13,367.75
PAAG_00173	electron transfer flavoprotein subunit alpha	23,108.45
PAAG_04931	electron transfer flavoprotein subunit beta	14,274.43
PAAG_02265	mitochondrial F1F0 ATP synthase subunit	16,924.33
PAAG_01078	alternative oxidase	15,371.76
	**Methylcitrate cycle**	
PAAG_04549	mitochondrial 2-methylisocitrate lyase	11,769.04
PAAG_04550	2-methylcitrate synthase *	13,649.55
PAAG_04559	2-methylcitrate dehydratase *	13,423.09
	**Propionate metabolism**	
PAAG_02163	propionyl-CoA carboxylase	11,642.86
	**Pentose-phosphate pathway**	
PAAG_04444	transketolase	9554.287
PAAG_04166	transaldolase	9647.347
	**Glyoxylate cycle**	
PAAG_04542	malate synthase	7841.01
PAAG_07786	acetyl-CoA acetyltransferase	9100.539
	**Energy conversion**	
PAAG_03631	12-oxophytodienoate reductase *	10,708.71

Functional classification by FunCat2, (http://pedant.gsf.de/pedant3htmlview/pedant3view?Method=analysis&Db=p3_r48325_Par_lutzi), * Proteins that formed multiprotein complex with *P. lutzii* ICL in a BN-PAGE assay. ** The confidence of each identification is evaluated by the estimated probability of the correct-match distribution.

**Table 2:** Validation of the chromatographic assay of ICL interacting proteins from mycelium, mycelium-to-yeast transition and yeast cells according to STRING.

Accession Number	Protein Name	Score
PAAG_04542	malate synthase *	0.998
PAAG_05048	3-isopropylmalate dehydratase large subunit ^#^	0.983
PAAG_03138	alanine-glyoxylate aminotransferase ^&^	0.943
PAAG_02682	fructose-1,6-bisphosphatase *	0.933
PAAG_08203	phosphoenolpyruvate carboxykinase ^#^	0.926
PAAG_07725	peroxisomal (S)-2-hydroxy-acid oxidase ^&^	0.911
PAAG_03793	dihydrodipicolinate synthase ^&^	0.900
PAAG_08075	citrate synthase *	0.899
PAAG_04550	2-methylcitrate synthase *	0.858
PAAG_06563	succinate/fumarate mitochondrial transporter ^&^	0.835
PAAG_02653	acetyl-coenzyme A synthetase ^&^	0.827
PAAG_04549	mitochondrial 2-methylisocitrate lyase *	0.819
PAAG_02361	D-amino-acid oxidase ^&^	0.816
PAAG_04751	hypothetical protein ^&^	0.800
PAAG_11872	hypothetical protein ^&^	0.785
PAAG_03275	molybdenum cofactor sulfurase ^&^	0.757
PAAG_08859	peroxisomal multifunctional enzyme ^&^	0.735
PAAG_00053	malate dehydrogenase *	0.733
PAAG_02585	triosephosphate isomerase *	0.678
PAAG_01995	fructose-bisphosphate aldolase 1 *	0.676
PAAG_06224	carnitine O-acetyltransferase ^&^	0.663
PAAG_00726	pyruvate carboxylase ^$^	0.662
PAAG_02732	2-oxoglutarate dehydrogenase E1 ^§^	0.649
PAAG_04851	osmotic growth protein ^#^	0.649
PAAG_07729	isocitrate dehydrogenase *	0.638
PAAG_11349	peroxisomal biogenesis factor 6 ^&^	0.626
PAAG_01015	Hexokinase ^&^	0.624
PAAG_08449	malate dehydrogenase *	0.621
PAAG_0058	fumarate hydratase *	0.607
PAAG_04856	acetyl-CoA hydrolase	0.605
PAAG_08057	cytochrome c oxidase *	0.602

* Proteins specific from yeast protein extract; ^#^ Proteins common to mycelium, mycelium-to-yeast transition and yeast protein extracts; ^&^ Proteins specific from STRING database; ^$^ Proteins common to mycelium-to-yeast transition and yeast.

## Supplementary Materials

The following are available online at https://www.mdpi.com/2309-608X/6/4/309/s1, Figure S1, ICL interacting proteins according to STRING database. STRING database identified 31 *P. lutzii* ICL partners. The cut-off score selected was less stringent (0.6) in order to verify if the number of PPIs from the STRING database that corroborates our lab-bench result increases. Table S1, Crystal structures and scores of the ICL interacting proteins modeled by the I-TASSER server.
