# Supplementary material for: Interaction of Isocitrate Lyase with Proteins Involved in the Energetic Metabolism in Paracoccidioides lutzii
Source: J Fungi (Basel). 2020 Nov 23;6(4):309. doi: 10.3390/jof6040309 (PMC7712234; doi:10.3390/jof6040309)
Supplement: Supplementary file 1 [file jof-06-00309-s001.pdf]

Supplementary Table 1 - The PDBs of relevant structures that interact with *Pb*ICL and their significance (C-score confidence and TM-score) used for decoys.

Accession	Protein name	PDB (decoy)	C-score*	TM-score**
PAAG_01995	fructose-bisphosphate aldolase	3QM3	1.02	0.81
PAAG_02869	phosphoglycerate kinase	1QPG	1.85	0.98
PAAG_06380	pyruvate kinase	1A3W	1.09	0.86
PAAG_08468	glyceraldehyde-3-phosphate dehydrogenase	4O59	1.05	0.86
PAAG_11169	enolase	1EBG	1.66	0.94
PAAG_08449	malate dehydrogenase	1MLD	1.02	0.83
PAAG_04550	2-methylcitrate synthase	5UQO	1.84	0.91
PAAG_04559	2-methylcitrate dehydratase	5MVI	1.03	-0.81
PAAG_06309	enoyl-CoA-hydratase	3Q0J	1.06	0.82
PAAG_08203	phosphoenolpyruvate carboxykinase	1AQ2	-2.06	0.47
PAAG_05249	alcohol dehydrogenase	1O9J	1.85	0.98
PAAG_08163	fumarylacetoacetase	4QKU	-1.58	0.94
PAAG_02633	ribose-phosphate pyrophosphokinase	4LYG	0.99	0.56
PAAG_04291	nucleoside diphosphate kinase	6JOH	1.57	0.93
PAAG_2633	ribose-phosphate pyrophosphokinase	4LYG	1.09	0.78
PAAG_03447	acetyl-CoA acetyltransferase	6L2C	-0.25	0.68

* Estimation of the quality of predicted models; ** Structural similarity score to measure the accuracy of structure modeling.

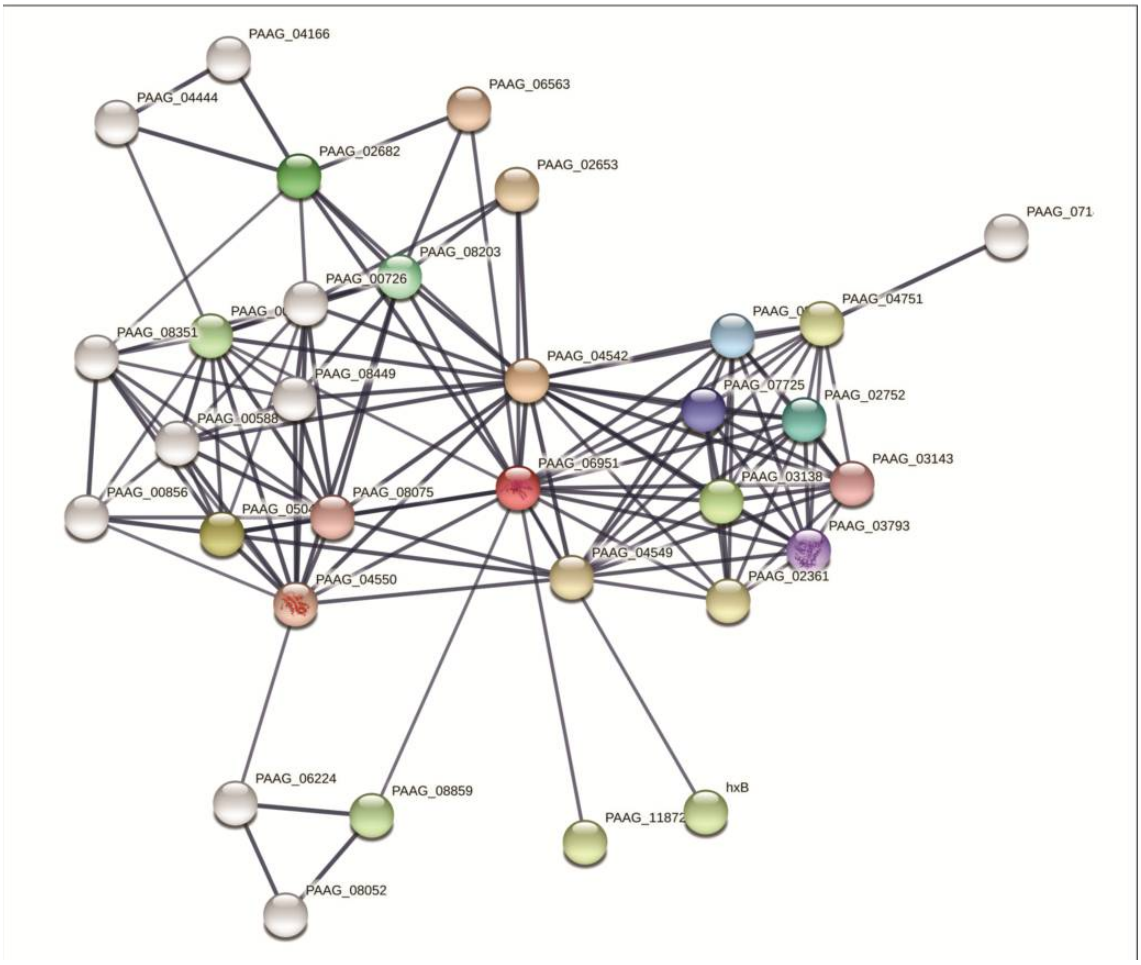

Figure S1, ICL interacting proteins according to STRING database. STRING database identified 31P. lutziiICLpartners. The cut-offscore selected was less stringent (0.6) in order to verify if the number of PPIs from theSTRING database that corroborates our lab-bench result increases.
